# Interactions and Pastoralism Along the Southern and Southeastern Frontiers of the Meroitic State, Sudan

**DOI:** 10.1007/s10963-015-9089-1

**Published:** 2015-11-28

**Authors:** Michael Brass

**Affiliations:** Institute of Archaeology, University College London, 31-34, Gordon Square, London, WC1H 0PY UK

**Keywords:** Sudan, Indian Ocean trade, Jebel Moya

## Abstract

The Nilotic Meroitic state, in what is now the Sudan, existed from the late fourth century BC until the mid fourth century AD. It has come to be regarded in recent years as an African segmentary state with a prestige-goods economy, less centralised than, for example, Egypt, with direct control by the ruling family diminished outside the Shendi Reach (central Sudan). Outbound trade from its capital Meroe included ebony, elephants, gold, iron, ivory and ostrich feathers. Trade routes criss-crossed the desert and extended down the Nile river to Greco-Roman Egypt, as well as through Red Sea ports to several Middle Eastern destinations including Egypt. Using the southern and southeastern reaches of the Meroitic state as a case study, I argue that to conceptualise the frontier peripheries of early states as borders is to misunderstand their internal dynamics (movements of people, fluid social networks and regional exchange systems). Each region had its own distinctive form of power relations. Examining how communities in these frontier zones were constituted, inscribed their identities in the landscape and facilitated trade in relation to the core of the Meroitic state in the Shendi Reach draws attention to the fluidity and continual renegotiation of state–pastoral relations.

## Introduction

**Fig. 1:**
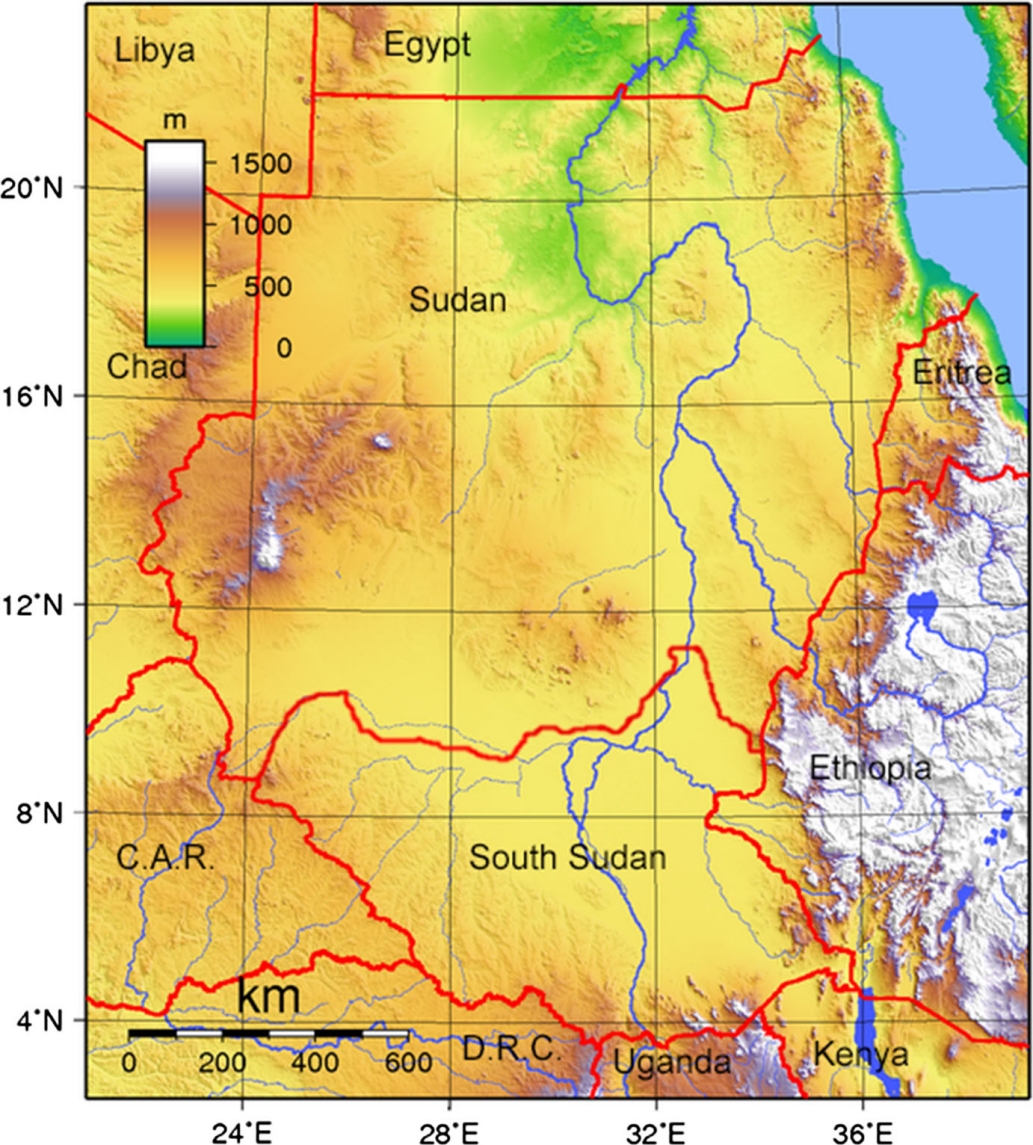
Map of Northeast African countries

Archaeological studies of pastoral polities and their social structures have traditionally been peripheral to discourses about socio-political complexity. This paper aims to contribute to the growing interest in the archaeology of frontier zones and mobility in early Northeast Africa (Fig. 1) (Barnard and Wendrich 2008; Brass 2014; Osman and Edwards 2012; Sadr 1991; Welsby 2001; Wengrow et al. 2014). It adds to a growing body of data that recognises that the differential forms of pastoralism are not a passive response to ecological or state-imposed conditions. Instead, the scope and structure of pastoral societies are the result of dynamic, contextually specific interactions between state and local (nomadic, semi-sedentary and sedentary) actors (Honeychurch 2013).

**Fig. 2:**
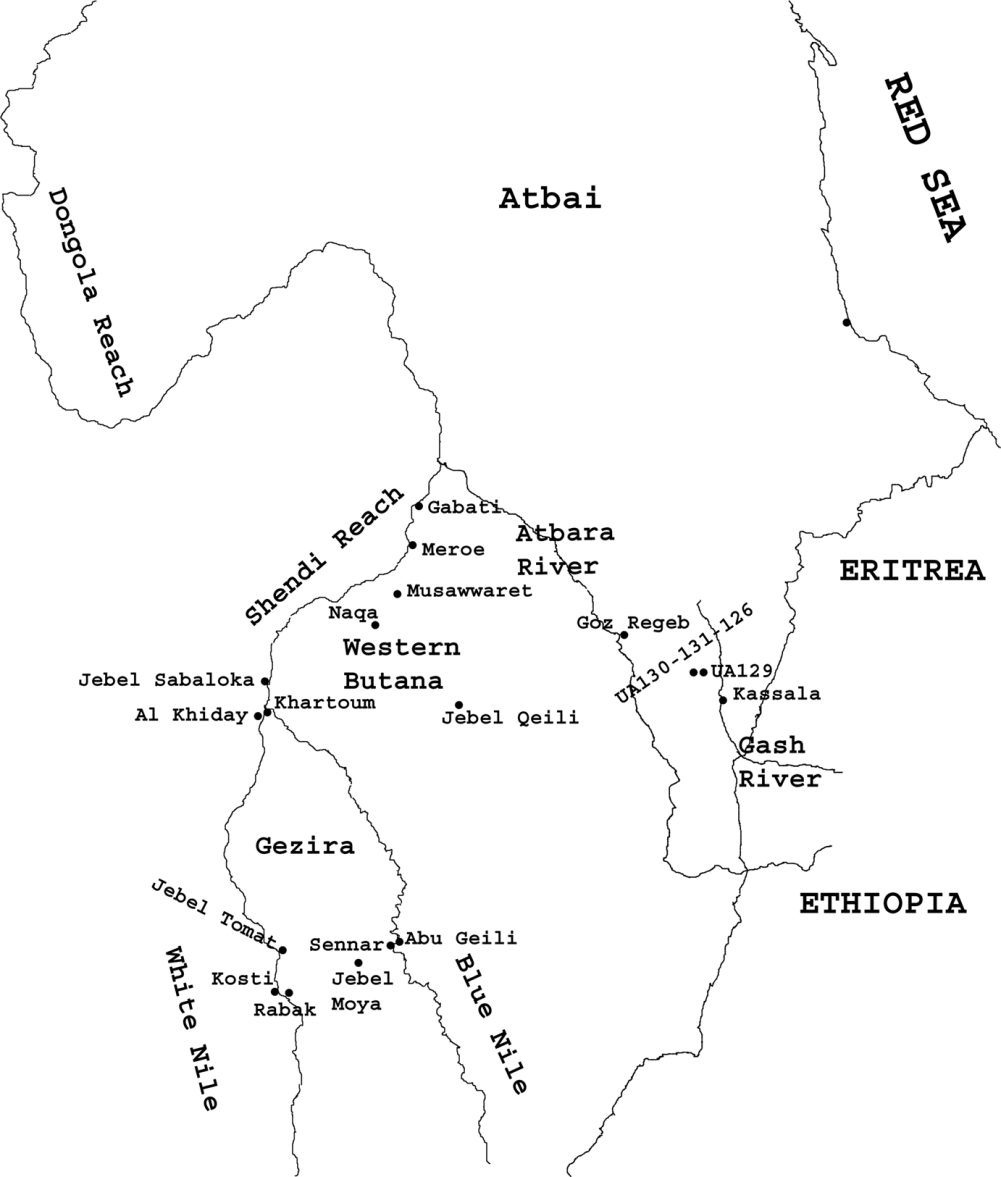
Map of the Sudan showing the sites in the Shendi Reach, southern Gezira and southern Atbai

The Meroitic state stretched from Lower Nubia down through the Shendi Reach (Central Sudan) and past the 6th Cataract of the Nile (Fig. 2). The data discussed in this paper comes firstly from my recent work, which re-evaluated the occupation of Jebel Moya (the largest pastoralist burial complex in sub-Saharan Africa, situated in the southern Gezira Plain, south of Khartoum), and secondly from past and ongoing work in the neighbouring western Butana and southern Atbara regions. I examine these areas—crossroads of trade and influences between state actors, agro-pastoralists and pastoralists—in order to understand how changing social, economic and power relations were conceptualised. I outline how they were characterised by various forms of social integration and economic exploitation within a trade network linked to the Meroitic elite in its Shendi Reach heartland.

## The Appearance of Nomadic Pastoralism in the Sudan

There have been many attempts to define the term *pastoralism*. It has been held up as an economic adaptation (Khazanov 1994) or cultural phenomenon (Ingold 1985). Alternatively, Emanuel Marx (2006) questioned whether the term is useful at all, on the basis that pastoral specialisation at the interface with settled communities is but one expression of the specialist diversity inherent within the latter’s economy. Pastoralists may become sedentary, that is, change their economic base either permanently or semi-permanently, without losing their cultural identity (Szuchman 2009). Anthropologically, the cultural, economic and social differences between groups of pastoralists have discouraged pigeon-holing them. Labels can apply at multiple scales, to whole communities, segments or internal status groups (Bollig et al. 2013, p. 23). Lack of hierarchical authority does not, *contra* Salzman (1999), mean that stateless societies such as the Nuer should be lumped together in a catch-all category of ‘egalitarian’: pastoral societies have never fitted neatly into debates on socio-economic and political complexity (Honeychurch 2013).

Instead, examination of pastoral societies should consider how power is diffused. Wealth-levelling mechanisms do not sufficiently disguise or muzzle social and societal variation. Power is a bi-directional continuum, where highly-specialised pastoral economies can develop rapidly under favourable conditions (McIntosh 1993, 1998) and can re-diversify just as rapidly (Bollig et al. 2013, p. 20). Rather than query whether hierarchical inequality is present (see Bourguignon and Greenbaum 1973), it is more productive to determine multiple elements of social differentiation and how these are reflected in socio-economic and political organisation, which may or may not be in a state of change (Borgerhoff Mulder 1999).

In the Sudan, dedicated animal husbandry by pastoral communities is said to have arisen during the first millennium BC from more generalised food economies (Table 1) (Linseele 2010), notwithstanding the debate over the timing and nature of the first domesticated cattle and ovicaprines in Northeast Africa (Brass 2007, 2013; Di Lernia 2006; Gautier and Close 1987; Gautier et al. 2001; Grigson et al. 2000; Hassan et al. 2000; Smith 1986; Stock and Gifford-Gonzalez 2013; Wendorf and Schild 1998; Wendorf et al. 1984). Nomads are defined here as specialised mobile stock keepers, who maintain relations with sedentary groups for the purpose of obtaining foodstuffs such as grain which they do not produce themselves. Whereas in the Near East the main pastoral animals were ovicaprines and, particularly, camels, in Northeast Africa cattle predominated; more specifically, zebu cattle, which were introduced in the mid first millennium BC from the Middle East and which can travel greater distances with lower food and water requirements (Linseele 2010, p. 47). Sadr (1991) has proposed a similar date for the advent of nomads, arguing that the first archaeologically visible nomadic entity in Northeast Africa is the Hagiz Group in the southern Atbai, southeastern Sudan (Table 2).

**Table 1:** A brief chronological summary of the history of the Nile Valley and Gezira, Sudan (From Garcea 2011; Gatto 2006; Honegger 2012; Kabaciński 2011; Sadig 2010; Salvatori and Usai 2007; Wengrow et al. 2014)

Period	Central Sudan	Upper Nubia	Lower Nubia
8300–6000 BC	Mesolithic	Mesolithic	pre-Neolithic Khartoum variant
6000–5500 BC	Mesolithic	Early Neolithic	pre-Neolithic Khartoum variant, Early Abkan
5500–5000 BC	Mesolithic	?	Khartoum variant, early Abkan
5000–4500 BC	Early Neolithic	Middle Neolithic A	Khartoum variant, developed Abkan
4500–4150 BC	Early Neolithic	Middle Neolithic B	Khartoum variant, developed Abkan
4150–3100 BC	Late Neolithic	?	Khartoum variant, developed Abkan, Early A group
3100–2600 BC	?	pre-Kerma	pre-Kerma, A-group
2600–1500 BC	?	Kerma	Kerma, C-Group
1500–1000 BC	?	Egypt—New Kingdom conquest	Egypt—New Kingdom conquest
1000–800 BC	?	Third intermediate period	Third intermediate period
800–300 BC	Napatan	Napatan	Napatan
300 BC—AD 350	Meroitic	Meroitic	Meroitic

**Table 2:** A chronological summary of the southern Atbai of the southeastern Sudan (From Manzo 2012a, b; Winchell 2013)

Period	Southeastern Sudan
6000–5000 BC	pre-Saroba
5000–4000 BC	Malawiya Group (Saroba phase)
4000–3800 BC	Malawiya/Butana transition
3800–2700 BC	Butana Group (Kassala phase)
2700–1700 BC	Gash Group (Kassala phase)
1700–500 BC	Jebel Mokram (Kassala phase)
500 BC—AD 500	Hagiz Group (Jebel Taka phase)

In the Sudan, the autonomous role of nomads, and of pastoralists more generally, as both agents and non-passive recipients of social change is a subject studied by few (Barnard and Magid 2006; Gatto 2011, 2014; Hafsaas 2006; Manzo 2012b; Sadr 1988). The greatest difficulties lie in identifying the presence of nomads or other pastoralists at a site, or gauging the extent of their involvement in craft and exchange networks. Archaeological markers could be a series of shallow graves with distinct patterns of accompanying burial assemblages and/or animals such as cattle or goats (Brass and Schwenniger 2013); bioanthropological data such as isotopic analysis or dental caries (Usai et al. 2014; Hutton MacDonald 1999); or tumuli (Gatto 2014; Hafsaas 2006; Welsby 2001). Spatial and statistical analysis of graves can yield data on the social structure of the population, as manifested in the mortuary realm (Brass 2014). In addition, there may be texts detailing trade or other interactions, written by state actors. The difficulty in interpreting these diverse material records lies in the nature of the interaction between nomads, pastoralists and states, which is based on fluid and varied combinations of distributed authority, exchange, forms of mobility and social use of the landscape (Fowles 2002; Honeychurch 2013; Szuchman 2009, p. 8). For this paper, this difficulty is resolved by a closer examination of the socio-political structures of and of inter-relationships between the Meroitic state and the southern Gezira and southern Atbai regions.

## The Socio-political Nature of the Meroitic State

Early states in the Saharan and Sahelian belts were comprised of diverse political systems, whose sources of power and systematic relationships were counter-poised between population segments and resulted in different trajectories of power and development (MacDonald, in press; McIntosh 1998, 1994, 1999a; Southall 1988). Resting and drawing upon these different resources, such polities ranged from centralised states to segmentary societies and frontier communities. However, for Northeast Africa, the centralised economic and ritual model derived from studies of Ancient Egypt has traditionally been applied to the Meroitic state (Dann 2009; Fuller 2003; Török 1995). This state evolved out of the preceding Napatan state in the mid fourth century BC, when the elite capital relocated to Meroe (Adams 1977; Grzymski 2004; Török 1997), which has a long pre-Meroitic occupation sequence stretching back to the early first millennium BC (Shinnie and Anderson 2004).

More recently, attempts have been made to place the Meroitic state in a Sahelian context by drawing upon Southall’s ethnographic model of segmentary lineage societies (Edwards 1996, 1998b; Fuller 2003; Southall 1988, 1999). In such a system, ritual and political influences have different spheres of control: ritual activities in the peripheral areas are in constant flux, while political authority is centred on the core domains of the territory, held in place by checks and balances of both ritualised sanction and institutionalised interdependence. Regardless of the validity of applying the concept of a segmentary society to the Meroitic state, when there are a myriad of social systems in the Sahel Belt (McIntosh 1999b), the application of anthropological data to the issue of the socio-economic structure of the Meroitic state moves the discussion away from the previous over-reliance on Egyptianised interpretative perspectives of burial and monumental structures. There is now a recognition of the fluid, contextual nature of exploitation and power relations, both between and within diverse geographical and administrative regions.

More broadly, formal political ties were further cemented by conferring titles and court privileges on regional elites, and by intermarriage. In return, tribute flowed through exchange corridors along the Nile to the political heartland in the Shendi Reach. Long-distance exchange and trade was largely conducted under state auspices, with royal gift exchange and royal embassy exchange being the mechanisms through which ‘exotic’ and prestige items were redistributed amongst the elite. Long-distance trade was focused on ‘luxury’ products. In essence, there was a prestige-goods economy, in which prestigious objects played a central role in social transactions and the marking out of status, and provided a medium through which high-level socio-political relations were forged (Edwards 1996).

A combination of the historical influence of Egyptology on Meroitic studies, the northern concentration of early rescue expeditions, and a focus on the main political and religious centres of the Nilotic-oriented early civilisations, has meant that little large-scale, systematic attention has been paid to the southern and southeastern frontiers outside the confines of the Nile (Ahmed 1984; Bradley 1992; Brass and Schwenniger 2013; Kleppe 1986; Manzo 2012b; Marks and Mohammed-Ali 1991; Sadr 1991). Supporting a model of a variety of socio-economic cultures in a mosaic of trade and political alliances in the frontier zone of the southern Gezira is the account by the Roman writer Seneca (Nat. Quest. VI 8, 3) of the first century AD Roman Emperor Nero’s expedition to trace the Nile upstream. The expedition’s likely purpose was to investigate new trading sources for the wider Roman Egypt—Meroitic state—Indian Ocean trade network in operation at this time. Seneca relates that the Meroitic ruler issued a letter requesting the local elites to grant unspecified assistance to the expedition’s members (Welsby 1996). Therefore, there appears to be textual data indicating the Meroitic elite’s engagement with the communities to the south; the evidence for contact to the southwest has been much more contentious.

There is a long-running debate about whether any eastern cultural influences can be detected in the architectural and mural styles of the buildings and towns in and around Meroe. The predominant view seems to be predicated in part on a belief that the Meroitic state did not participate directly in the Indian Ocean trading system, but instead utilised Egypt as its intermediary for trade with the world beyond Africa. Consequently, many scholars are inclined to see suggestions of Arabian or Indian elements (personnel, stylistic or artefactual: Arkell 1951; Hofmann 1975) in the Shendi Reach as effects of Egyptianised Hellenism only (Wenig 1978; Zabka 1975). Arguments in favour of Meroitic outreach into the ancient world beyond the African continent include earlier Kushite horse-trainers (occupational specialists) in ancient Assyria (Heidorn 1997), and Meroitic emissaries at Rome (Shinnie 1967) and Constantinople (Peake 2010). Given that Kushites also travelled to the heart of the Persian Empire, and that the Roman Empire engaged in trade with southern Arabia and India, it seems implausible that the Meroitic state had no cultural contact with southern Arabia and India. This, then, was the premise for Haaland’s (2014) new exploration of the relationship between Meroe and the Indian Ocean trading system.

In recent years, new evidence has come to light which justifies Haaland’s revival of Hofmann’s postulation of a cultural influence or exchange: firstly, Sidebotham’s (2011) publication of Indian trade goods found at Berenike; and secondly, Abdu and Gordon’s (2004) analysis indicating that Meroitic iron-making techniques were paralleled most closely by Indian techniques, rather than by those of the Mediterranean or sub-Saharan Africa. Berenike was one of the Egyptian ports from which trading expeditions were sent down the Red Sea to the Arabian Peninsula and India. However, a University College London—Qatar team is conducting a comprehensive examination of the ironworking remains and technology in and around Meroe (Humphries and Rehren 2014), and it remains to be seen whether they corroborate this conclusion of Abdu and Gordon’s. Ultimately, Haaland (2014) hypothesises that there was a system of mobile occupational specialists, moving over vast distances, which was responsible for the diffusion of cultural ideas and crafts. She also argues for the existence of an elite gift exchange involving prized ivory, textiles and spices, which leave few archaeological traces along the overland trading routes except where accumulated in large quantities, as at Red Sea ports. Haaland’s contention is supported by Larsen’s (1987) argument for an extensively-used overland trade network whose existence is known almost exclusively from texts in early second millennium BC Anatolia and Mesopotamia, while Phillips (1997, p. 443) notes that a hypothesis of intermediaries transporting goods which leave little archaeological trace ‘should not be minimized or dismissed simply because there is little or no archaeological evidence for it’, despite the paucity of texts mentioning overland routes.

### Western Butana

Outside the confines of the Nile Valley in the Shendi Reach, the Western Butana is arguably the only area under the personal control of the Meroe royalty (Ahmed 1984; Edwards 1996, p. 90), with the southeastern boundary in the vicinity of Jebel Qeili, c. 148 km southeast of Khartoum, marked by a rock inscription of King Shorakaror dating from the second half of the first century AD (Edwards 1996, p. 12; Török 1997, p. 205). This inscription has been interpreted as commemorating the pacification of, and establishment of rule over, this area (Török 1997, p. 466–467).

Monsoonal rain agriculture—notably sorghum (Fuller 2014)—was practised at this northernmost edge of the Sahelian savannah belt (Ahmed 1984), while pastoral activities also occurred (Bradley 1992). Like the southern Atbai (below), it is better known archaeologically than the neighbouring southern Gezira, although it remains under-explored and under-excavated in comparison with Lower and Upper Nubia, due to the aforementioned historical circumstances (Bradley 1992; Edwards 1989, 2007). Evidence for the extension of direct elite control over the Western Butana is provided by the construction of *hafirs* (dams), which, along with the settlement-distribution pattern seen more broadly in the region, may reflect a seasonal mobile pastoral exploitation with a trade and food-exchange relationship between the pastoralists and settled communities along the edge of wadis (Bradley 1986, 1992).

The archaeological focus in the Western Butana remains very site-specific, and is centred particularly on the monumental architecture of Musawwarat es Sofra, Basa and Naqa. These sites—like the small temples at other localities which are classed as religious ‘Lion temples’—have centres for the god Apedemak (Edwards 1999, p. 26). Apedemak was a war god and his cult was associated with the Meroitic royal elite. Large and smaller temples in his service are known in the Western Butana, lending weight to the hypothesis of elite oversight through which resources were channelled and redistributed outwards to the rest of the state (Edwards 1996, p. 27).

Pawel Wolf, heading the German expedition at Hamadab (Western Butana, Sudan) and Wuqro (Tigray, northern Ethiopia), sees no archaeological evidence for direct contacts between the Meroitic state and the Indian Ocean trade network. He does, however, believe that there was more indirect east–west contact than is traditionally accepted (north–south contacts down and up the Nile are well-established). He proposes that the general cultural ideas of Meroe, expressed for example in the basic concepts of sacral architecture and manner of construction, may have been influenced by ideas prevailing in Tigray at sites such as Yeha and Wuqro (Wolf 2014). The latter, in turn, came via southern Arabia. They all combined in the ‘Island of Meroe’ with ideas and patterns derived from the north, developing into an eclectic mix visible at Musawwarat es Sufra and Naqa.

Musawwarat es Sufra is viewed by Wenig (2001, p. 86) as a locality where Mediterranean traders could have purchased African elephants. According to this interpretation, the site’s Great Enclosure (c. 55,000 m^2^) could also have served as a training ground for the elephants. Representations of elephants show some adorned with cloth said by Haaland (2014, p. 665) to be found in India rather than in the Sudan, although this claim requires closer investigation. In both Egypt and the Napatan and Meroitic states, elephants were trained primarily by Indian *mahouts* (Kistler 2007). Haaland (2014, p. 668) believes that this is an example of occupational specialists moving between distant, culturally-separated lands to ply their trade, leading also to the diffusion of ideas.

More fieldwork is required to evaluate these hypotheses. What is clear though is that, as Edwards (1996, p. 26) says, ‘emphasis should be placed on the Western Butana as a zone of political interaction. More generally, the history of the Meroitic expansion into the Western Butana should be conceived of as an essentially political phenomenon’. The timing of the southwards Meroitic expansion from the end of the last millennium BC coincides with the expansion of Indian Ocean trade from Myos Hormos and Berenike (where numerous Indian textile remains have been found) in Egypt, down the Red Sea and across to India via the port of Adulis (Sidebotham 2011, 2014). Also at Berenike, Sidebotham (2014) has found a number of miniature stone offering-tables (religious), whose nearest parallels are with Nubia and Meroe. Meanwhile, the *Periplus Maris Erythraei* (*Circumnavigation of the Erythraean Sea*), a commercial shipping guide to the Red Sea—Indian Ocean trading network written in the mid first century AD by an unknown Greek merchant trader based in Egypt with first-hand experience, records contemporary imports to the southern port of Adulis, including textiles (Huntingford 1980). There was an annual peak of 120 Roman ships participating in the Red Sea trade in the first century AD. Control over the Western Butana would have facilitated overland travel from the Shendi Reach out through its wadis across the trade routes to the Abyssinian highlands and ports along the Red Sea.

### Geological Settings of the Southern and Southeastern Frontiers

Although ecological conditions are not the primary determinant of the form of pastoralism adopted in a particular region, an understanding of the environmental conditions of the southern Gezira and the southern Atbai is essential to re-evaluating how pastoral societies chose to adapt to their circumstances.

#### Southern Atbai

This region, the southernmost extension of the Eastern Desert, situated between the Atbara river and the Ethiopian Highlands, has a modern annual rainfall of 200–400 mm (Sadr 1991, p. 25). It is predominantly comprised of clay plains stretching to the beginning of the Ethiopian Highlands, cut through by the Atbara river valley and flanking badlands. It had a wetter climate until a drying trend began at the start of the Middle Holocene (3000–1000 BC), leading to the retreat of the flood regime and the gradual establishment of a savannah ecology (Sadr 1991, p. 30). As a consequence, the course of the Gash river shifted until, at the end of third millennium BC, it began to empty into an inland delta north of the town of Kassala, at the eastern edge of the plain and close to the geological start of the Ethiopian Highlands. Although the Gash river is seasonal, fed by summer rains, its water table remains high for the remainder of the year and is accessible via wells.

Over the millennia, evolving social structures permitted and incorporated increasing numbers of cattle as trading conditions and relations with neighbouring states changed. Karim Sadr describes the settlement of Kassala in the Gash Delta of the southern Atbai:

Culturally, Kassala is a frontier zone. Located on the boundary of highland Ethiopia and lowland Sudan, it has been successively occupied since 1840 by Turko-Egyptians, Mahdists, and Anglo-Egyptian and Italian forces … Besides Kassala Town and a few smaller communities around it, the only other major population centre in the region lies on the western bank of the Atbara at Khashm el Girba. The Atbara river forms a cultural boundary … The Gash river banks and its delta are the most fertile zones of the Southern Atbai … The Sharab el Gash (literally, wine or drink of the Gash), some 35 km south of Kassala Town, is the second most fertile zone of the Southern Atbai study area (Sadr 1991, p. 26–28).

This ecology also impacts the potential for early cereal domestication practices in the Delta (Beldados Aleho 2012; Winchell 2013, pp. 137–138, 140), a thorough discussion of which is beyond the scope of this paper. It is, though, the subject of ongoing investigation by a joint Institute of Archaeology (University College London)—Italian Expedition to the Eastern Sudan (University of Naples ‘L’Orientale’) project.

#### Southern Gezira

The fertile Gezira (Arabic for ‘island’) is a megafan built by the Blue Nile and criss-crossed by Late Pleistocene and Early Holocene channels (Williams 2009, p. 7). The Blue Nile is highly seasonal, whereas the White Nile has an almost constant flow throughout the year. The two meet north of Khartoum (Williams 2009, p. 3). During the Holocene, some Blue Nile palaeo-channels were partially—perhaps seasonally—active until as late as the fourth millennium BC (Williams 2009, p. 7). The present-day unregulated flood levels were in place by the mid third millennium BC, by which time the transition from Early–Middle Holocene swampy conditions to a mostly acacia tallgrass savannah, and subsequently semi-desert steppe, was also complete as the Inter-Tropical Convergence Zone retreated southwards. There was a brief interlude of wetter weather around the end of the second millennium BC, as determined by two OSL dates on sandy clay samples east of Jebelain at site S2 (Mawson and Williams 1984; Williams 2009; Williams et al. 2010). Swamp conditions continued at certain localities along the White Nile, such as Jebel Tomat, to the northwest of Jebel Moya, where they co-occurred with dry land in the early centuries AD (Clark 1973). Today, the nearest riparian swamps are over 12 km to the south.

In modern times, the mean annual rainfall recorded from 1921 to 1950 across the Gezira is approximately 400 mm for the isohyet line nearest to Jebel Moya—the largest pastoral cemetery in sub-Saharan Africa, dated to between the end of the first millennium BC and the mid first millennium AD (Brass and Schwenniger 2013). To put this into perspective, 164 mm was recorded at Khartoum over the same period (Williams et al. 1982, pp. 136–137). The southern Gezira can be sub-divided into swamp vegetation, riverine woodland, grassland and jebel vegetation. Below 400 mm there is a gradual transition from clay thornland alternating with grassland to semi-desert grassland (Mubarak et al. 1982). This places the Jebel Moya environs close to the transitional belts, both in modern times and probably during the Classic and Later Meroitic periods as well. Jebel Moya itself is a geological outcrop of the plain’s underlying Basement Complex through the Sandstone Formation and its overlay. It is comprised of granulitic rocks, which also occur at Jebel Saqadi some 20 km to the northwest (Vail 1982, pp. 54–55).

This geological context is important for reconstructing the environment of Jebel Moya (southern Gezira) and the localities of the southern Atbai. It also helps us to understand that (a) the foothills of Jebel Moya were a potential source of fresh water, as the Basement Complex broke through the overlying sandstone and gave access to its underground aquifer in a plain otherwise without permanent surface water, and (b) ecological conditions in both regions were sustainable for pastoral activity in the late first millennium BC to early first millennium AD.

## The Archaeology of the Southern Atbai

The Eastern Butana and the lands to the east and southeast of it make up the Southern Atbai or Southeastern Sudan. Its ceramic tradition—the Atbai Ceramic Tradition—shows an indigenous development that began as early as the sixth millennium BC and continued uninterrupted through to the Hagiz Group (Table 2) (Fattovich et al. 1984; Winchell 2013). The transition from the Late Gash phase to the Jebel Mokram phase occurred c. 1500 BC (Table 2) and is currently under investigation by the Italian Archaeological Expedition to the Eastern Sudan, led by Andrea Manzo. Although thick everted rims and thick and channelled wares became common, with rim decoration including spatula-impressed herringbone and other stylus- and comb-impressions, there is no discernible relationship between the Gash and Jebel Moya pottery (personal inspection, Naples 2013). It is during the Jebel Mokram phase that pastoralism increased in southeastern Sudan, through migration of pastoralists from Upper Nubia, with agricultural and pastoralist specialisation and separation of these activities within the societies (Sadr 1991). Agricultural activity is evidenced by the presence of both domesticated and wild sorghum (*Sorghum bicolor*) at Mahal Teglinos (Kassala, Gash Delta) during this period (Beldados Aleho 2012, p. 100). It is also during the Jebel Mokram period that increased numbers of Pan Grave artefacts are found in the Eritrean–Sudanese lowlands, which indicates intermediary contact with Upper and Lower Nubia, and also with Egypt via the Red Sea (Manzo 2012b, p. 78; Sadr 1991).

By the mid first millennium BC, the Jebel Mokram group had evolved into the Hagiz group (Jebel Taka phase). It has a markedly different set of ceramics, consisting of thick, heavily fibre-tempered (probably with chaff) pottery vessels, which were haphazardly combed on both the exterior and interior surfaces (Sadr 1991, Fig. 9; Winchell 2013, p. 16). There are matt impressed sherds in both the Nile Valley and southeastern Sudan at this time (Manzo 2011, p. 5). It has been suggested that the pottery from the Hagiz group shows slight linkages with the pre-Axumite from the Abyssinian highlands, towards which it was oriented (Edwards 1989, p. 37; Sadr 1991, p. 69). By the mid first millennium AD, the Hagiz group was replaced by other groups who no longer shared any of the ceramic traits associated with the Atbai ceramic tradition (Fattovich et al. 1984; Winchell 2013, p. 17). The distribution pattern of sites from this phase resembles that of the preceding Jebel Mokram phase: they are typically situated at the base of outcrops and in the open plains between Jebel Mokram to the east of Kassala and the Atbara river (Manzo 2012a, p. 105). An example is provided by the sites JM1 and JM2. JM1, located east of Jebel Mokram in the Kassala region and fairly close to the border with Eritrea, is a cluster of tumuli on flat ground. JM2 is close to a jebel (mountain) and also comprises a cluster of tumuli.

An apparent shift in subsistence from the agro-pastoralist economy associated with the Jebel Mokram group to a more nomadic existence associated with the Hagiz group is evidenced by an increase in the number and spread of low density sites (Sadr 1991; Winchell 2013, p. 17). Unlike the Jebel Mokram group, who herded cattle, sheep, and goats, the Hagiz group were said to have placed a greater emphasis on cattle herding and possibly depended on trade to obtain grain products (Sadr 1991, pp. 56, 59). More recently, evidence for a more nuanced interpretation of the economic pathways in the region has emerged at the settlement site UA 129, indicating that perhaps ‘the agropastoral model may have survived at least in the Gash Delta’ (Manzo 2012a, p. 105). Imprints of domesticated and wild sorghum were found on pottery sherds collected during surface surveys in 2010 and 2011 at UA 129 (Beldados Aleho 2012, p. 101). Outside the Gash Delta, nomadism predominated.

The dimensions of Hagiz group localities are vast, as determined by the 2010 and 2011 surveys of the Italian Archaeological Expedition to the Eastern Sudan: ‘The sites in this phase have extensions spanning from 800 to 600,000 m^2^ (60 ha). Most are characterised by an extension ranging between 10,000 and 40,000 m^2^’ (Zoppi 2012, p. 45). They are all low density locales. To date, it is unclear whether there are any thick cultural deposits. The known surficial deposits include little in the way of faunal remains, and what there is derives solely from small-sized cattle domesticates. Artefacts of any kind, bar the odd occurrence of grinding stones, are rare (Sadr 1988, p. 396).

Hagiz group settlement localities with nearby cemeteries are not unknown. Cemetery UA 130-131-126, for example, is so close to a settlement that they are considered one site (Manzo 2012a, p. 105). A small number of undecorated pottery sherds are said to show links to the Meroitic culture to the west (Fattovich et al. 1984, p. 182), but this claim requires re-investigation. It is to be hoped that ongoing research in the eastern Sudan and Wolf’s expedition in the ‘Island of Meroe’ will assist in clarifying aspects of the relationship between eastern Sudan and the Nile Valley. As Sadr outlines,

the case of the Hagiz Group does not fit the ecological theories for the development of nomadism [i.e. that] nomads are such because they have no access to arable lands. Yet, the situation in the southern Atbai of Period 4 clearly shows that the Hagiz nomads occupied practically the entire study area, including the most agriculturally fertile zones of the region: the Kassala sector … Before c. 750 BC, the Southern Atbai was a cultural and economic heartland. The Mokram Group of Period 3 had connections with the cultures of Nubia and the Eastern Desert of Egypt … During the second millennium BC, the Southern Atbai was a province of the land of Punt, trading partner to Pharaonic Egypt. After the early first millennium BC, this picture was completely reversed. Both the central Sudanese Nile Valley [the Napatan and successor Meroitic states] and the northern Ethiopian Highlands [pre-Axumite and Axumite centers] became political, cultural and economic centers. While these neighbouring regions enjoyed a major cultural florescence, the Southern Atbai became a cultural, economic, and political hinterland sparsely inhabited by the Hagiz Group nomads. The change in the status of the Southern Atbai occurred in the absence of any significant environmental changes. As a whole the region never became an ecologically marginal zone. Instead, it seems that during the first millennium BC, it had become a politically and economically marginal region. Thus, the development of nomadism in the Southern Atbai can perhaps be seen as a response to politico-economic changes rather than environmental ones (Sadr 1991, pp. 396–97).

Under this model, following that originally proposed by Sadr (1991), nomadism is first evidenced in the southern Atbai shortly after the establishment of firstly, the Napatan state (eighth century BC), and secondly, the contemporary pre-Axumite kingdoms in northern Ethiopia. However, I do not agree with Sadr’s notion that the region became economically and politically marginal to the Meroitic state, but neither do I argue that the group was incorporated into the Meroitic sphere of economic influence. What I propose is that movement of craftspeople and perishable trade goods from the port of Adulis on the southern Red Sea coast would have passed through the Abyssinian highlands, the Gash Delta and the southern Atbai *en route* to the Nile Valley, although admittedly there is nothing in the main ceramic assemblages of the Hagiz group to indicate connectivity with Central Sudan. There is, however, indirect evidence for contact between the Shendi Reach and the Eastern Desert. Such evidence includes (1) pottery with similarities to so-called Eastern Desert ware at Gabati in the Shendi Reach and at the Red Sea port of Berenike in Upper Egypt (Barnard and Magid 2006; Magid 1998; Manzo 2004), and (2) the possibility that Tabot in the Red Sea Hills was utilised as a trading station for outbound Meroitic goods in the Indian Ocean trade system (Barnard and Magid 2006; Magid 1998).

Support for the idea of multiple trade routes across the desert comes in the form of two Soba-ware bowls from the fifth century AD found at Aksum (Phillips 1997, p. 455), and a note from a sixth century AD Byzantine writer, Procopius of Caesarea, summarised and discussed by Phillips, who stated:

it was then a journey of thirty days from Aksum to Aswan for an ‘unencumbered’ traveller, a situation probably not very different throughout the millennia before, and this and other texts tell us it was no more than between eight and fifteen days travel to Aksum from Adulis. This presumably main overland route from the Red Sea coast, through the Aksum and Kassala areas, probably via or near the Gash (Mareb) and Atbara rivers through to the eastern bank of the Nile valley around the Kurgus area and then up to Aswan—bypassing the Nile river almost entirely—seems to have been a well-travelled trading route for a long period of time. Whilst archaeological confirmation is tenuous, capillary and feeder routes must also have existed (Phillips 1997, pp. 440–441).

What the information from the admittedly later source Procopius may also indicate is that there could have been another route out of the Shendi Reach through the western Butana into the southern Atbai and up to more northerly Red Sea ports via the Eastern Desert, which may explain the presence of Eastern Desert ware at the Shendi Reach. There is also an image of the Meroitic ruler Shorkaror (c. AD 20–30) incised on a boulder *en route* at Jebel Qeili in the Butana. Shorkaror is holding several captives, suggestive of either an attempt at pacification (Török 1997, p. 466) or at retribution in order to ensure the continued viability of the trade route. The latter is more likely, as Meroitic objects have been found as far east as Goz Regeb (Andrea Manzo, pers. comm. 2015). Consequently, the Hagiz group may have adopted nomadism not because the southern Atbai was a marginal area, but rather because its economic and trade adaptability and fluidity permitted them to take advantage of and influence changing circumstances along their borders.

## The Archaeology of the Southern Gezira

To date, the Gezira, and in particular the southern Gezira, is distinguishable from the Nubian provinces of the Meroitic state not just by burial practices but also by the paucity of status artefacts manufactured in the production centres of its Shendi Reach heartland. While these regions were certainly culturally and politically distinct, the status of communities outside Meroitic control, and the degree and type of their access to the exchange networks that would have allowed them to obtain such objects, must also be considered (Edwards 1996; Osman and Edwards 2012). Furthermore, how the status of individuals was represented by the organisation of the burials themselves and the removal from active circulation of artefacts holds potential for determining the burial ideologies of these communities. Alternatively, any lack of status objects may have been because such items were not used in such ways in mortuary rites (Chapman and Randsborg 1981, p. 13; Goody 1962, p. 71), which, coupled with the lack of adequate survey and excavation of identified sites, would produce a distorted picture of the nature and subsequent use of the trade relationship between the inhabitants of the periphery and the Meroitic centre.

Unfortunately, absence of comprehensive surveys means there are insufficient excavated and securely-dated sites to allow us to adequately address such problems as the Meroitic exchange systems extending into the southern Gezira. Most of the known sites were discovered by Wellcome’s expedition in the first half of the twentieth century (Crawford and Addison 1951), supplemented in the early 1970s and mid 1980s by J. D. Clark (Clark 1973; Clark and Stemler 1975; Clark et al. 1973) at Jebel Tomat and by Randi Haaland (Haaland 1984, 1987) at Rabak, while Sandro Salvatori and Donatella Usai (2014) are excavating at Al Khiday in the northern Gezira. The Meroitic-era remains from Al Khiday are radiometrically dated to between the first century BC and the second century AD, contemporary with Jebel Moya. While the northern Gezira is regarded as being within Meroe’s direct sphere of influence, if not political control, Jebel Moya, is the largest and best-excavated site in the southern Gezira; it yields new insights into the nature of its pastoralist settlement and mortuary practices (see Brass 2014, bringing together and differentiating the occupational sequence in the southern Gezira).

### Jebel Moya

**Fig. 3:**
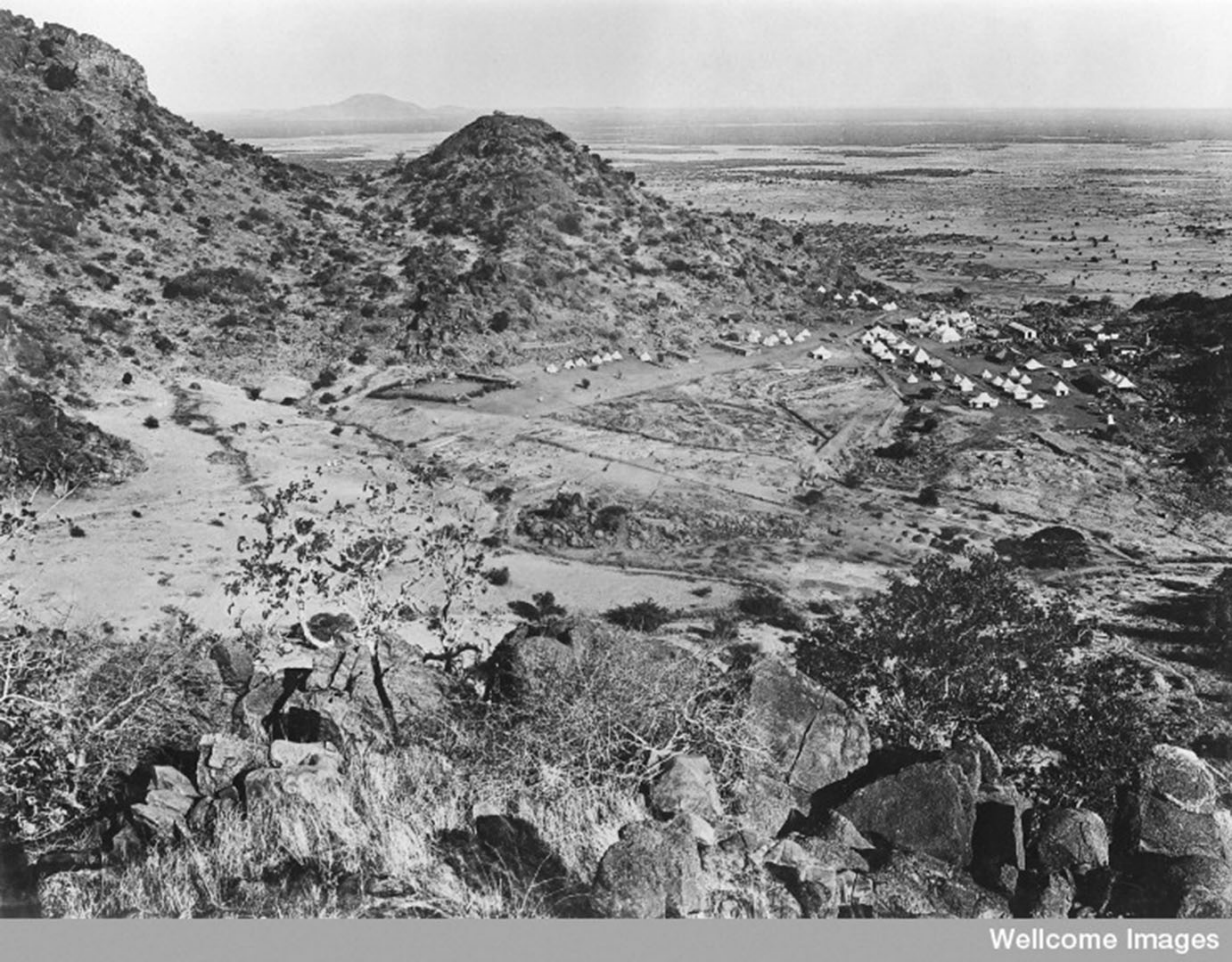
Excavation camp at Jebel Moya, 1912–1913. Copyright Wellcome Trust

The Jebel Moya massif is situated c. 250 km south-southeast of Khartoum, and approximately 30 km west of the modern town of Sennar (Blue Nile) (Fig. 3). The excavated valley, formally known as Site 100 and spanning 10.4 ha, is located in the northeastern sector of the massif. Sir Henry Wellcome, the founder of the Wellcome Trust, initiated excavations in January 1911, and these continued over four field seasons until April 1914, shortly before the onset of the First World War (Addison 1949). Around a fifth of the estimated 10.4 ha was excavated, yielding 2882 recorded graves and pits, of which 2791 were excavated by Wellcome. These include 3135 human burials, of which 1108 are associated with burial goods. No post-war excavations were undertaken by Wellcome, who passed away in 1936. The archaeological (Addison 1949, 1956) and bioanthropological (Mukherjee et al. 1955) remains were analysed and subsequently published.

Although a few studies have since been undertaken (Caneva 1991; Clark and Stemler 1975; Gerharz 1994; Haaland 1981, 1984, 1987; Irish and Konigsberg 2007), none have comprehensively re-evaluated the archaeological archive. The records and osteological remains are curated at the Duckworth Laboratory (University of Cambridge), while the photographic archive, the diary of the second season’s field excavator (Oric Bates), and some notes are at the Griffiths Institute (Oxford); representative samples of the pottery are at the British and Petrie Museums (London); and a small sample of pottery and assorted artefacts are at the Museum of Archaeology and Anthropology (University of Cambridge). Small numbers of artefacts are distributed among other museums in the UK and elsewhere. The focus of my doctoral work was to re-evaluate the existing UK-based archival and curated records and materials for evidence of social organisation, elucidating the nature of the socio-political order in the southern Gezira Plain. This was achieved through focusing on obtaining a secure chronology (Brass and Schwenniger 2013) and investigating the social aspects of the burial assemblages (Brass 2014) to enable the development of more sophisticated hypotheses about the development of Jebel Moya and the processes affecting its cultural evolution.

In order to arrive at a secure chronology for Jebel Moya, I re-evaluated the pottery collection at the British Museum (Brass and Schwenniger 2013). This entailed single and co-occurring attributes of tools, their motor actions and location, and the luminescence dating of six pottery samples by the Research Laboratory for Archaeology and the History of Art (Oxford University). Three periods of occupation have been determined (Table 3):

**Table 3:** OSL results on the dated samples from the British Museum Jebel Moya collection (From Brass and Schwenniger 2013)

Laboratory code	Palaeodose (Gy)	Total dose rate (Gy/ka)	OSL age estimate (years before 2012)	Calendar date
X5291	9.71 ± 1.49	5.52 ± 0.38	1760 ± 295	40 BC—AD 550
X5292	16.33 ± 3.64	5.03 ± 0.33	3245 ± 755	1985–475 BC
X5293	7.19 ± 1.20	4.82 ± 0.32	1490 ± 270	AD 255–790
X5294	17.38 ± 2.30	5.06 ± 0.33	3435 ± 260	1680–1165 BC
X5295	17.96 ± 2.14	5.53 ± 0.37	3250 ± 445	1680–790 BC
X5296	7.58 ± 2.58	4.90 ± 0.33	1545 ± 535	70 BC—AD 1005

Period 1 dates to the late sixth or early fifth millennium BC. Whether its communities were hunter-gatherers or had a mixed economy is unknown. Period 2 is bracketed between the mid second millennium and the first half of the first millennium BC. It is unknown what economic practices were followed, but it is assumed, on the basis of mixed economies to the north, that they were agro-pastoral or pastoral. Finally, Period 3 is from the first century BC until the mid first millennium AD, and it is to this pastoral phase that the vast majority of the burials are assigned (Brass and Schwenniger 2013).

The placement of the site’s different phases of occupation in secure temporal contexts allowed for informed social analysis of change over time. Together with the results of the re-sexing of the extant human remains at the Duckworth Laboratory (undertaken by the then-curator Mercedes Okumura) and the compilation of a new Register of Graves from the surviving expedition’s records, it facilitated the identification of structuring principles of the mortuary remains at Jebel Moya using spatial and statistical analyses (Brass 2014).

**Fig. 4:**
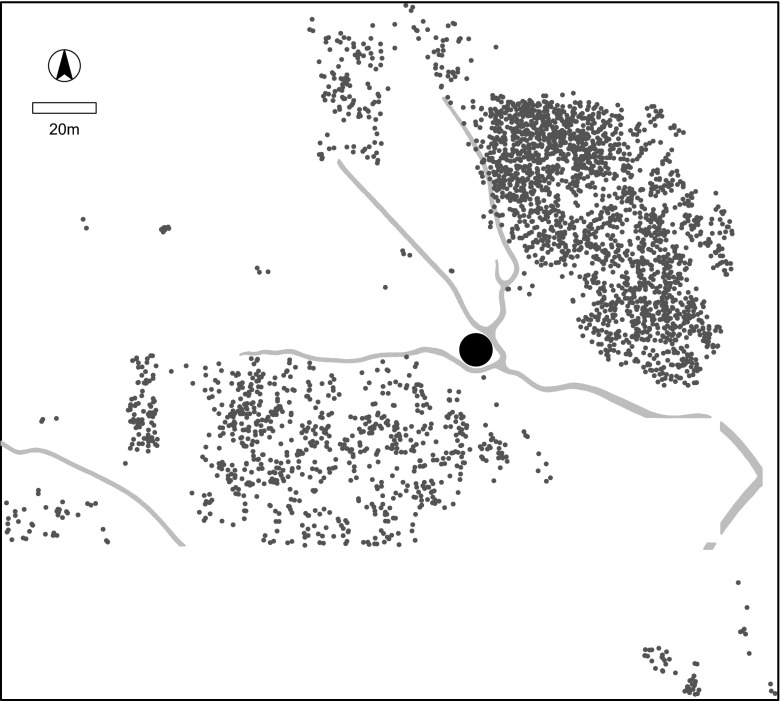
Map showing the spread of the burials in the Jebel Moya valley. In the *centre* is a large rock outcrop, while the *lines* represent water courses (From Brass and Schwenniger 2013)

It is also necessary, however, to establish the extent to which habitation remains were present alongside the mortuary remains, in order to more fully understand the nature of the site (Table 4). Four geological strata were present across the valley and labelled A–D in descending order by the excavators. The original ground surface of Stratum C was used as the reconstructed datum point by Addison (1949, pp. 26). Attempts to re-evaluate the nature of habitation activities within the valley for the first time since Addison (1949) are hampered by the absence of surviving field notes detailing the type and stratigraphic positioning of the stated presence of floors and other features outside the southwest sector of the excavated area of the valley (Fig. 4). In the southwest, the ground surface at the time of excavation was on average 210 cm above the surface of Stratum C, and the surface of Stratum D c. 75 cm below. Of the claimed human-made features, those below the surface of Stratum C consist of stone flooring; a probable hearth; three hardened mud floors, with one exhibiting signs of post-holes and a second with a hearth; and two hearths. All bar the stone flooring and one hearth were on or in the immediate vicinity of the surface of Stratum D, which is consistent with habitation activity possibly contemporary with Phase 1 and/or (if including the stone flooring and hearth) Phase 2.

**Table 4:** The known features recorded as evidence for human habitation activity in the Southwest sector, their re-evaluated designation and depth above or below the surface of Stratum C

Original	Re-evaluation	Level above	Level below
Small patch burnt clay flooring	Probable hearth	195 cm	
Stretch hardened paving	No information to re-evaluate	180 cm	
1st flooring	Calcium carbonate formation	c. 157 cm	
2nd flooring	Calcium carbonate formation	c. 150 cm	
3rd flooring	Calcium carbonate formation	60 cm	
Post-holes in 3rd flooring	Post-holes in calcium carbonate formation	60 cm	
3 ovens	3 ovens	Between 1st and 3rd ‘floorings’	
Hardened mud	Mud plaster from wattle-and-daub structure(s)	Unknown	
Mud pavement	Unverifiable	50 cm	
Baked clay floor	Unverifiable	25 cm	
Stone flooring	Stone flooring		20 cm
Hardened burnt clay	Probable hearth		25 cm
Burnt floor paving	Unverifiable		25 cm
Floor paving	Unverifiable		35 cm
2 places of burnt earth	Probable hearths		c. 75 cm surface of D stratum
*Tukl* floor	Unverifiable, apart from it being hardened mud		Surface of D stratum
Post-holes in tukl floor	Post-holes in hardened mud		Surface of D stratum
Hardened floor of round dwelling with burnt earth in middle	Possible dwelling floor with hearth		Surface of D stratum
Red-ware pot sherd floor	One or more vessels crushed under pressure		Surface of D stratum

The ground surface here at the time of excavation was 210 cm above C surface

Above the surface of Stratum C, the claimed first–third floorings were naturally occurring calcium carbonate features. Three ovens were also unearthed within the same levels, c. 157–160 cm above Stratum C. They, together with post-holes within the third ‘flooring’ feature, attest to habitation activity. There is also mud plastering from a wattle-and-daub structure, and a claimed stretch of hardened paving; however, there is no sketch or photograph to verify the latter feature. The mud plaster is from Stratum B but its exact stratigraphic positioning cannot be reconstructed. The latest activity was a small patch of burnt clay flooring at 195 cm above C surface, which is in Stratum A and towards the end of the period of mortuary activity at the site. The stratigraphic positioning of these remains correlates with the pottery sequence whereby Period 3 pottery is present in the upper strata in greater numbers than Period 2 pottery (Brass 2015). Overall, the weight of evidence strongly suggests that there are habitation remains in association with all three phases of occupation.

The mortuary assemblages consist of a wide range of artefact categories, but the numbers of actual artefacts, other than lip studs and beads, are low. Consequently, I focused upon ‘potential sources of origin for the raw materials from which the items were manufactured [which] can be reasonably deduced and acted upon for analysis. Inverse Distance Value (IDV) is therefore defined here as the weight (value) assigned to a material which diminishes as the distance from the area/region of origin decreases’ (Brass 2014, p. 431). In order to examine whether there might be differences in the way individuals were interred, the presence and types of artefact categories were examined for each individual re-sexed by the Duckworth Laboratory and calculated as IDV (Table 5). The pattern of artefact deposition between adult females and males has a degree of uniformity.

**Table 5:** Breakdown by sector of the IDV per Duckworth Laboratory aged and sexed burials

	Number of individuals	Mean	Median
*Southwest*			
Adult. Female	69	3.35	2
Adult. Male	100	1.45	0
Adult	222	2.4	1
Young adult	7	3.57	2
Juvenile	7	2.4	0
*West*			
Adult. Female	0	0	0
Adult. Male	1	0	0
Adult	0	0	0
Young adult	0	0	0
Juvenile	0	0	0
*Northwest*			
Adult. Female	16	3.5	0.5
Adult. Male	24	1.17	0
Adult	47	1.94	0
Young adult	3	1	0
Juvenile	2	1.5	1.5
*East*			
Adult. Female	33	1.52	0
Adult. Male	40	1.13	0
Adult	90	1.42	0
Young adult	3	9.67	0
Juvenile	5	0.4	0
*Northeast*			
Adult. Female	84	0.89	0
Adult. Male	90	1.03	0
Adult	233	0.98	0
Young adult	12	0.33	0
Juvenile	8	0.63	0

The ‘adult’ category numbers include the totals for the female and male adult individuals

IDV was also used to conducted spatial analysis. When pair correlation function analysis was run against the IDV for all individuals, only the northeast sector, where there is the greatest density of burials, exhibited spatially distinct patterns (Brass 2014). The 27 highest value burials formed a spatial neighbourhood. Within this, there were fewer than expected poorer burials within a c. 20 m radius of any individual rich burial. In other words, there appears to have been some kind of social prohibition on burying within 20 m of these richest burials, while their individual locations were permanently marked in unknown ways. The rich burials have no distinguishing construction features; like the poorer ones, they are in relatively shallow, non-lined graves with no superstructures.

Neither is there is anything bioanthropologically distinctive about these 27 individuals. They formed part of a distinctive bio-cultural homogeneous grouping centred on the southern Gezira within a greater Northeast African sphere (Irish and Konigsberg 2007). Their economic base has been identified as pastoral, through Rachel Hutton MacDonald’s (1999) dental anthropological studies of occlusal macrowear, buccal microwear and carious lesions, taking into account feature density due to age and wear. The archaeological presence of cattle figurines and the inclusion of only cattle and dog either accompanying human burials or, in the case of cattle, also as separate burials, lends support to this conclusion.

Having habitation and mortuary remains together within a high but confined valley above the surrounding plain would have created social collective memories of the dead (Bollig 1997; Sterner 1995). The articulation of these memories and their intersections can reflect (1) family, lineage or communal interaction with the deceased, (2) social roles and responsibilities, (3) personal possessions decommissioned from the realm of the living, or a combination thereof. The spatial neighbourhood in the northeast sector is paralleled by the sector possessing the greatest number of burials, which may be due to an effort by (1) competing or lesser lineages to associate themselves in death with the dominant lineage, thus constructing a subservient undercurrent of affiliation within the broader ideological remit of group collectivity, or (2) related lineages emphasising their relationship in death with the dominant lineage through deposition in the same vicinity. These mortuary rituals co-existed with the living in the same valley in a prominent mountain range rising above a flat plain. How the pastoral occupants moved in their landscape outside the valley is a question which has not previously been explored.

### Other Gezira Sites in Relation to Jebel Moya and Meroe

As part of the ongoing Jebel Moya project, Patrick Quinn conducted the first ever petrographic analysis of Jebel Moya pottery sherds from the Petrie Museum’s (University College London) collection at the request of the author. This new data has a direct bearing on how the pastoralist inhabitants of Jebel Moya moved in and utilised features of the immediate and surrounding landscape, and thereby helps elucidate differences between pastoralism in the southern Gezira and the southern Atbai.

**Fig. 5:**
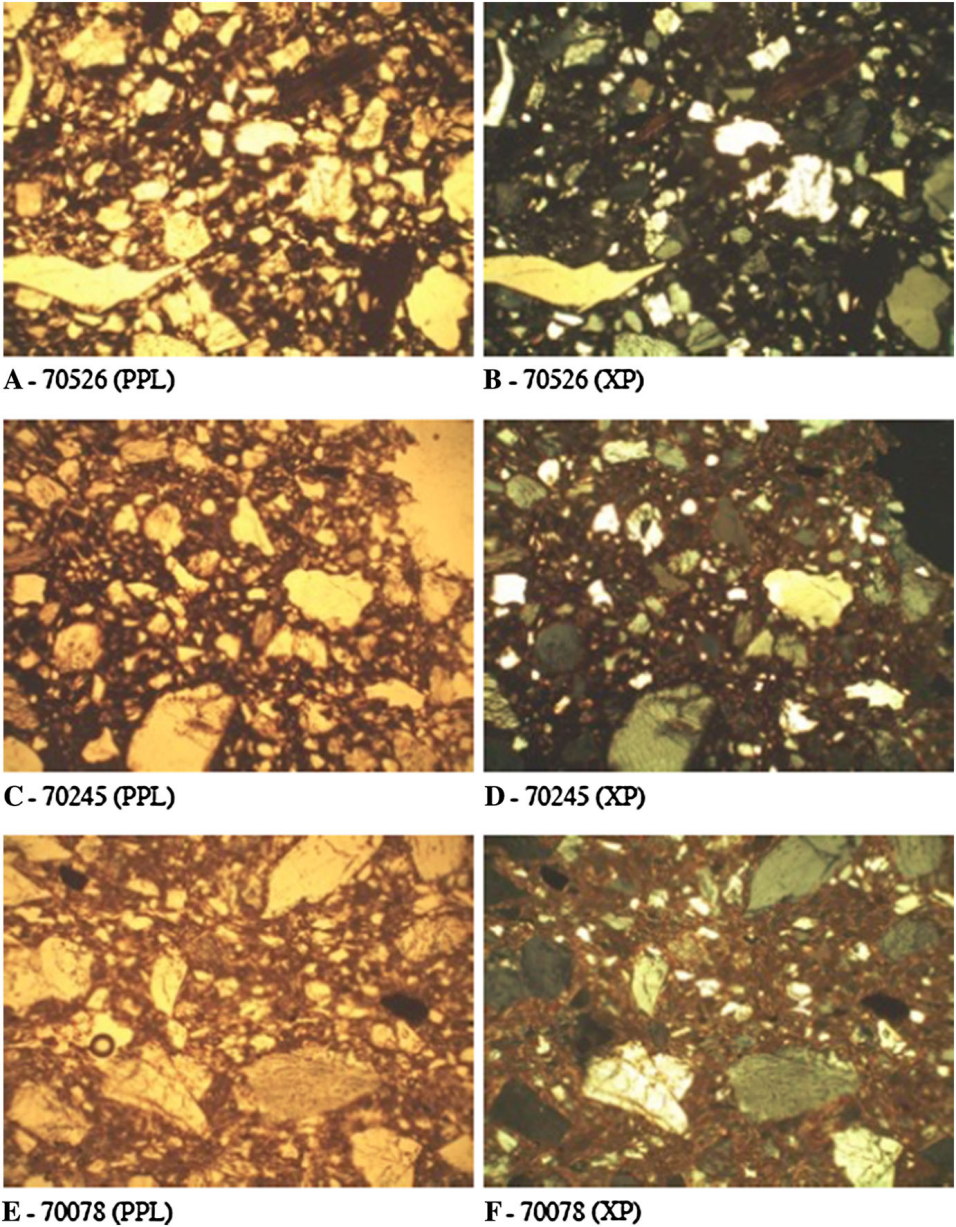
Thin section photomicrographs of three Jebel Moya sherds from the Petrie Museum collection dating to Period 3. *XP* crossed polars, *PPL* plane polarised light. Image width = 2.9 mm

All three sherds analysed are from Period 3 (Fig. 5). The thin sections were characterised petrographically under the polarising light microscope and interpreted in terms of their constituent raw materials and manufacturing technology. The likely source(s) of raw materials for ceramics were identified by comparison with a geological map and report of the study area (Williams and Adamson 1982).

The three sherds have a common petrographic composition, characterised by abundant, poorly-sorted, generally angular inclusions of quartz and feldspar in a non-calcareous clay matrix. The angular, poorly-sorted nature of the inclusions and the absence of material ascribable to another source rock strongly suggests that a residual or minimally-transported clay source could have been used for the ceramics. This clay may have been refined in some way, such as by the removal of coarse particles or plant matter, but there was little other processing. There is no evidence, either in terms of the composition or texture of the inclusions, for the addition of temper to the clay paste.

The inclusions and voids are randomly oriented, though sample 70078 may contain relic coils suggestive of the method used to form the vessel from which this sherd originated. Sample 70528 was fired in a reducing atmosphere, whereas samples 70078 and 70245 were well oxidised and moderately well oxidised respectively. The optical activity of the clay matrix in samples 70078 and 70245 suggests that firing was < 850 °C. The dark clay matrix of sample 70528 makes the observation of optical activity difficult. However, several amphibole inclusions in this sample have a brown colour, perhaps suggesting a firing temperature of > 750 °C.

Granite occurs within the Basement Complex that underlies central Sudan, and crops out in several places, including the region between Rabak and Sennar where Jebel Moya is located. Vail (1982, pp. 55) reports that granites intrude into charnockite at Jebel Moya and nearby Jebel Saqadi, c. 20 km to the northeast. The granite at Jebel Saqadi is composed mainly of equigranular quartz, orthoclase, plagioclase, perthite with biotite and rare amphibole. This matches the parent rock interpreted for the clay source used for the manufacture of the Jebel Moya ceramics. Based on this comparison with the general geology of central Sudan, it appears likely that the three ceramic samples analysed from Jebel Moya in this report were locally made. Further interpretation will be possible when comprehensive sampling is undertaken for both localities.

There is additional archaeological evidence for the exploitation of Jebel Saqadi. Saqadi was excavated by Duncan Mackenzie at the request of Sir Henry Wellcome in February 1913. A stone wall enclosure of 25 × 20 m was uncovered, inside which was a red mudbrick structure in the uppermost of three layers (Crawford and Addison 1951, p. 111). Trenches and pits were excavated along the inside and outside of the walls of the enclosure. Sherds were collected from inside and outside the structure. Most were hand-made and either black with red-infilled decoration or light brown in colour. The finer sherds were red-ware or black-polished, while the coarse sherds were also black or red. Other colours were present in limited numbers. Many but not all of the pottery forms are claimed by Crawford to have similarities with those of the agro-pastoral village Abu Geili (below), along the Blue Nile, while a sherd from the foot of the mountain was said to resemble sherds from Jebel Moya’s Period 3 assemblage (Crawford and Addison 1951, p. 121). Wheel-made pottery is present but in numbers described as ‘very low’, and only a small (unspecified) portion of it was painted (Crawford and Addison 1951, p. 120). No hand-made pottery is reported as painted. The painted sherds are said to be different from both Meroitic and Jebel Moya painted pottery (Crawford and Addison 1951, p. 121). It is, however, unknown whether any of the remaining wheel-made pottery was locally manufactured or imported from areas to the north. Other finds include pottery disks or ring fragments, stone rings, armlets and mace-heads, which Crawford claims are similar to their counterparts at Jebel Moya; these remains, present at the British Museum (London) and the Museum of Archaeology and Anthropology (University of Cambridge) have not been re-examined to verify these claims.

Overall, there appears to have been significant socio-economic interaction between the peoples of Jebel Moya and Jebel Saqadi, which also stretched to Abu Geili (below). However, whether the peoples at Jebel Moya and Jebel Saqadi were both pastoralists is a question which cannot be answered at present. The inhabitants of Jebel Saqadi should not be uncritically described as non-pastoralists based on the presence of the mudbrick building, since permanent or semi-permanent structures are known archaeologically and ethnographically from pastoral (including nomadic) societies (Honeychurch 2013). What is clear is that, at a minimum, the Jebel Moya–Jebel Saqadi–Abu Geili axis had close socio-economic interconnections.

Abu Geili is located c. 30 km east of Jebel Moya along the banks of the Blue Nile. The archaeological site comprises a village and a cemetery which was also excavated by Wellcome’s expedition in 1914. The cemetery was dated to the Funj Sultanate of the Middle Ages. The excavator, O. G. S. Crawford, dated the village to 200 BC—AD 600, based on the nature of the artefacts and types of pottery, which span the Classic, Late and post-Meroitic periods (Crawford and Addison 1951, p. 11). The village’s date has been re-confirmed by the AMS dating of a lump of charred sorghum grains and spikelets in the University College London Institute of Archaeology’s collections to 1790 ± 40 bp (Fuller 2014, p. 169), calibrated to AD 127–344 (OxCal Intcal13 95.4%). Overall, there is some possibly imported wheel-made pottery, but the majority is locally hand-made and distinct from the Jebel Moya assemblages (Crawford and Addison 1951, p. 42), although some Jebel Moya wares were present (Brass and Schwenniger 2013, p. 439). These Jebel Moya wares are from Period 3 and consist of black polished, incised, cord-wrapped stamped and comb-stamped wares, as illustrated by Crawford and Addison (1951, Plate XXXVIIIB). However, I observed additional unpublished Jebel Moya-like sherds amongst the curated Abu Geili assemblages during a cursory look while re-examining the Jebel Moya pottery assemblages at the British Museum in 2012. Thus the original pottery assemblage contains more pieces than acknowledged from communities living in the vicinity who utilised the Jebel Moya cemetery.

Nearby, the cemetery at Sennar was discovered in 1921 on the east bank of the Blue Nile, 2 km south of Abu Geili. The remains include carnelian, Lydian stone, faience, glass and quartzite beads, faience figurines (*Bes* and *Amun* ram with the sun disc), pottery and bronze vessels (Addison 1950). There were a limited number of hand-made pottery forms similar to those found at Jebel Moya, indicating trade exchange and at least some contemporaneity, although the pottery was not illustrated and has not yet been located for re-examination. The hand-made pots are regarded as local forms (Addison 1950). The wheel-made red-ware was probably imported from Meroe via the exchange networks, as were the jars of common Meroitic form. Some forms of the bronze vessels have been found at Meroe West Cemetery (Addison 1950). Similar carinated bronze bowls occurred in elite burials at Meroe, reinforcing the elite exchange network hypothesis of Edwards (1996). The latter also raises the strong possibility that there is an undiscovered accompanying settlement, now inundated by the Sennar Dam, which functioned as an outpost for Meroitic administrative and trading purposes. Such an outpost would have served a vital function in an area that had potential to act as a capillary conduit for the supply of slaves, ivory, ostriches, horn, gold, skins, elephants and leopards from the African interior for trading onwards down the Nile and to Egypt and India via the Red Sea (Haaland 2014, p. 653).

The increase in direct Roman commercial interest in the Red Sea coast arose after Augustus Caesar established control over Egypt in AD 31; the aim was to reduce, or take control of, the trade monopoly from the Arabian Peninsula states. The trading routes encompassed the southern Red Sea as far south as East Africa and round the Arabian Peninsula through to India. The *Periplus Maris Erythraei* records that ivory was brought to the port of Adulis via Aksum and its hinterland. African ivory was regarded as being of superior quality to its Indian counterpart. The *Periplus* also states that ‘all the ivory from beyond the Nile [is brought to Aksum] through the district called Kueneion, and thence to Adouli [Adulis]’ (Huntingford 1980). Kueneion has been hypothesised to be in the area of ancient Sennar, c. 350 km east of Aksum (Kirwan 1972, p. 166; Phillips 1997, pp. 450–451). The mention of the area ‘beyond the Nile’ should not be regarded as odd, as the Roman Emperor Nero sent an expedition to explore the origins of the Nile and the possibility of trade further up the Nile; this expedition ventured south from the branching of the Nile near Khartoum and from the southern Gezira to the start of the Sudd swamplands. More evidence though is required to substantiate or disprove this old hypothesis locating Kueneion on the banks of the Blue Nile.

Another grave was subsequently found in 1925 on the west bank by Sennar. All but one of the 40 pots were black ware with no external decoration, unlike the Jebel Moya black ware (Addison 1950). Addison claims there are affinities with the ceramics at Abu Geili, where Jebel Moya pottery types have been found, making the sites at least partly contemporary. The grave does not contain any distinctly Meroitic pottery but three more graves were found nearby by Arkell, one Funj, one post-Meroitic and one Meroitic, suggesting the former presence of a small cemetery in the vicinity, which was damaged or destroyed by riverine action (Addison 1950).

A mere 4 km downstream from Sennar Dam, two cemeteries have been identified at Karim’s Garden, consisting of 19 burials (Cemetery A) and 1 grave (Cemetery B) and accompanying burial assemblages and surface finds. The artefacts from Cemetery B contain pottery dated to the Meroitic (Edwards 1991, p. 46). There are many wheel-manufactured pottery vessels in addition to hand-made forms, with the other known locality of smaller wheel-made forms being Sennar East. Cemetery A’s pottery, however, is post-Meroitic, with vessels of similar shape, such as long-necked jars, gourd-shaped bowls and bevel-rimmed bowls known from Sennar to as far north as Kadada in the Shendi Reach (Edwards 1991, p. 46).

Another site in the southern Gezira is Jebel Tomat, to the northwest of Jebel Moya. It was excavated by J. Desmond Clark’s team in 1973 (Clark 1973; Clark et al. 1973). Its final and agro-pastoral occupation phase was radiocarbon dated to between the early first and the end of the fourth century AD (Clark and Stemler 1975), which makes it contemporaneous with the mortuary activity at Jebel Moya.

Subsequently, in the past three decades, there have been brief and somewhat haphazard surveys and excavation attempts. Between November 1986 and January 1987, Osman collected some artefacts while gathering samples for his doctorate on Late Quaternary palaeochannels in the southern Gezira. The pottery comes from the palaeochannel system running ‘north of the Managil Ridge through Nueila El Ugda, Goz Sheikh Mansour, and [joining] the White Nile at el Gutena, Garasa, and Wad El Zaki’ (Caneva and Osman 1990, p. 27). The sherds were examined by Caneva (1990), who ascribed them to the Mesolithic (dotted wavy line), the equivalent of Jebel Moya’s Period 1 pottery (Brass and Schwenniger 2013), and to the Meroitic and post-Meroitic periods. Meroitic (zoned) pottery has also been found at Dirwa, Diwaihia and Silaikab.

Additional limited survey work has resulted in more evidence for Meroitic trade down the Blue Nile. An isolated grave to the northeast of Sennar was dated to the Meroitic by David Edwards (Edwards 1991). South of Sennar is Dinder, where settlement and grave remnants were found in a mound in August 1997 (Ahmed and Ahmed 2004). The shape of the grave and the bodily orientation could not be determined, and no superstructure was evident. Four burials were excavated and numerous others recorded as visible during limited excavations. Pottery from Dinder and the village of El Sereifa just to its north is burnished black ware with limited incised decoration mainly on the rim. Interestingly, there were a couple of blackened sherds amongst the Jebel Moya Period 3 collection at the British Museum. Such pottery has traditionally been regarded as late Meroitic or early post-Meroitic (Ahmed and Ahmed 2004, p. 187), which would fall within the OSL range attained for Period 3 at Jebel Moya.

Further downstream at Qoz Nasra, near Marangan village, surface finds encompass the Meroitic to modern periods. At Umm Sunt, slightly upstream from Qoz Nasra, also on the west bank of the Nile, 19 of an approximate 45 burials have been investigated, and a Meroitic to post-Meroitic date assigned to the artefacts (Fernández et al. 2003).

Meanwhile, to the southwest of Jebel Moya is Aba Island, situated near the right bank of the White Nile, with Rabak on the east bank (Adam 2013). Survey work has found numerous pottery sherds with comb stamping, comb incisions, incised lines and zoned motifs, which point to a late first millennium BC or early–mid first millennium AD attribution; no Jebel Moya sherds are present in the few published photographs (Adam 2013, p. 146).

Meroitic items have also been found as far south as Kosti, to the southwest of Jebel Moya on the banks of the White Nile (Eisa 1999); near Grisly village; at El Getina, Dinder, and Wad Sheneina; between El Getina and El Kawa (e.g. El Teresab, Hashaba, Ni’ma and Wad el Zaki); and at El Tersab, 24 km south of El Getina, on a plateau containing Meroitic and Christian-period burials (Ahmed and Ahmed 2004; Eisa 1999; Fernández et al. 2003). These localities remain to be comprehensively excavated and published. An incised scarab (‘God Amun gives life like Re’) was found at El Kawa, and is said to have been made during the Napatan period (Eisa 1999, p. 367) but its date of deposition remains unclear. Objects (iron bracelets, pottery, etc.) speculated to be Meroitic in date but not in nature have been found south of Kosti and in western Sudan (west of the banks of the White Nile), but have yet to be adequately dated. Furthermore, while the site of Rose 5 along the Upper Blue Nile, which displays both settlement and burial activity, is potentially extremely informative as a link between the southern Gezira and South Sudan, neither comprehensive analysis of the pottery and lithic assemblages nor radiometric dating has been undertaken (Bashir et al. 2012).

Conversely, the northern Gezira Plain most probably fell under an unknown form of Meroitic rule during the Classic and Late periods. The construction of the graves at Site 16-D-4 (Al Khiday 2, on the west bank of the White Nile) shows Meroitic influence with a bedrock-incised chamber off the circular or ellipsoidal pits accessed by a shaft (Usai et al. 2014, p. 187). Like Meroitic burials at El Geili to the north there is a west–east orientation of the burial chamber and the grave, while the individual is contracted and positioned facing north with the head to the west; finally, a stone pillow is sometimes present (Usai et al. 2014, p. 193). The contrast with Jebel Moya, with its shallow sandy burials without stone linings or permanent superstructures, has been taken to mean that the funerary practices at Site 16-D-4 were influenced by their counterparts in the Shendi Reach, and that the area was within a sphere of political control by Meroe (Usai et al. 2014, p. 195), as previously postulated by Eisa (1999).

The southern Gezira, therefore, formed a dynamic zone of interaction on the Meroitic state’s southern frontier, with settled and semi-settled agro-pastoralists, at least one Meroitic station at Sennar, and a large pastoral occupation at Jebel Moya. The peoples were likely an integral component of the Meroitic trade network both down the Nile and—if the interpretation of the *Periplus* identifying Sennar is correct—across the Butana and the Eastern Desert, including the southern Atbai and Aksum, to the Red Sea. The social configuration at this time included the emergence of an elite at Jebel Moya, detectable in the mortuary realm through the spatial neighbourhood in its northeast sector. Differences in social organisation between these communities and those in the Meroitic political heartland can be elucidated by briefly outlining the mortuary behaviour exhibited at non-elite cemeteries in the Shendi Reach.

## Shendi Reach: The Heart of the Meroitic State

Two large, non-elite Meroitic cemeteries have been uncovered south of Meroe in the Shendi Reach and may relate to riverine settlement, though no associated settlement remains have been found and it is unclear whether such nucleated cemetery activity was the norm or the exception for the region. They are Kadada (Geus 1984; Lenoble 1994; Reinold 2008) and Gabati (Edwards 1998a; Judd 2012). At the Gabati cemetery (whose Meroitic phase dates from the second century BC to the end of the second century AD, and post-Meroitic phase from the start of the fourth century AD to the end of the eighth century AD), 63 of 74 identified Meroitic graves were excavated, out of a total of 124 burials. The remaining 50 burials (all excavated) were post-Meroitic and Medieval.

The average density was 2 graves per m^2^, which is a significant contrast to Jebel Moya where the density reached a maximum of 10 per m^2^, with 205 graves in square J.9, K.10 in the northeast. No offering tables or inscriptions were present. Graves with mudbrick and stone platform superstructures were rare, not just at Gabati but for non-elite tombs generally in the Shendi Reach outside of Meroe (Edwards 2004, p. 175): Of the 63, only 4 retain traces of a black sandstone chipped superstructure encased by mudbrick. This contrasts with the more frequent appearance of such structures in Lower and Upper Nubia to the north of Shendi Reach. Edwards speculated that the other graves may have been marked by a low sand mound. No such superstructures have been identified at Jebel Moya, where the pastoralists’ graves were oval or rectangular without burial shafts. Two of the superstructures at Gabati have traces of a probable chapel on the east side. The four superstructure burials contained chambers accessed by ramps. By contrast with the variable orientation of the Jebel Moya graves, the bodies at Gabati were more uniformly orientated east–west (in superstructures, an orientation more consonant with ‘classic’ Meroitic burials: Adams 1977, p. 374) or north–south (for the remainder at the end of sloping ramps in transverse chambers), potentially implying a more uniform local ideology. Additionally, a larger proportion of human burials contained pottery: 62.9% (Gabati) to 2.41% (Jebel Moya), with both wheel- and hand-made forms present.

Although these types of large ‘rural’ cemeteries are non-elite, they may have been nevertheless socially restricted in some way, as fewer have been found than would be expected if they reflected the normative funerary practice for the entire non-elite population. Edwards (2004, p. 175) has speculated that there may have been a second-tier of restricted access to inclusion in such grounds. Similar burials have been found to the south of the Shendi Reach, at Gereif East in the northern Gezira along the Blue Nile (Edwards 2004, p. 175). Few preserved tumuli are present in clustered cemeteries south of Khartoum, but some tumuli and other graves with similar artefact repertoires are known as far south as Sennar (Addison 1950; Al-Hakim 1979; Babiker 1984; Dixon 1963; Edwards 1991; El-Tayeb 1999; Marshall and Adam 1953).

South of Gabati and 35 km north of Omdurman is the cemetery of Bauda on the west bank of the Nile, just downstream of the confluence of the Blue and White Niles and near to the Neolithic site of Shaheinab. It spans over a thousand years, from c. AD 220 until 1504 and covers a vast area—1.5 × 0.6 km—with 118 burial mounds, of which 104 have been excavated (Babiker 1984, pp. 1, 134). The mounds are comprised of earth and gravel, low in height with variable diameters up to 15 m, and sometimes enclosed by a stone wall, something unknown at Jebel Moya. However, vast grave and tumuli fields are known from El Goi in the region of the 5th Cataract north of Berber, which the surveyors attribute to the Kerma and post-Meroitic periods respectively (Jesse et al. 2013).

The two Bauda mounds radiocarbon dated to the Late Meroitic were in the southern and northern sectors, indicative of a variable spread of graves not confined to specific areas for different time periods. In total there were 147 graves, of which 110 are Meroitic, 36 Christian and 1 post-Meroitic (Babiker 1984, p. 139). The bodies were contracted, head to the south on their right side and facing east in a standardised orientation not evident at Jebel Moya, which was more generally southwest to northwest. Although the bodily orientation to the south is different to that found at Gabati and likely indicative of local regional variation, the overriding ideology of maintaining a standardised pattern is evident. Unlike at Meroe, burial coffins are not present.

At Meroe, robber activity at many of the royal and non-royal graves has hampered efforts to determine the original burial posture and sometimes also the number of retainers (Shinnie and Anderson 2004). Its major cemeteries were located to the east and are called the Meroe South, West and North cemeteries. Edwards (2010) has recently attempted to seriate the pottery assemblages from the West cemeteries to derive a reliable ceramic chronology which could be applied both to the other Meroe cemeteries and more widely in the Shendi Reach, the Meroitic heartland. The elite tombs comprised a burial chamber with large superstructure, with a pyramid-shape indicating royal burial. There were also poorer graves, with chambers and narrow vertical shafts leading to them. All forms are very distinctive from those occurring at Jebel Moya, where the graves were shallow and without shafts or permanent superstructures.

Approximately 3 km south of Meroe is Hamadab on the east bank of the Nile river. It consists of two mounds. The northern mound formed the town (Wolf and Nowotnick 2006) while the southern mound was identified as the necropolis in 2005 as a result of robber activity. Many graves lacked a superstructure. Unlike at Kadero, they were richly furnished. The town and the necropolis have been dated to the Meroitic and post-Meroitic. Around 80 km upstream from Meroe is the town of Wad ban Naga, which has Meroitic and post-Meroitic graves and features and where excavations have just been re-opened (Onderka 2014).

Downstream from Meroe, and the confluence of the Nile with the Atbai River, is the newly-discovered cemetery of Berber, dating from the mid second century AD to the mid third century AD, where 34 tombs have been excavated in a rescue mission (Bashir 2010, 2013). The bodies in the burial chambers were flexed or semi-flexed, with the exception of BMC 8, which was extended and orientated south–north, and may have been buried in a wooden coffin (Bashir 2010, p. 71). Orientations were either north–south, south–north or east–west.

Upstream from Gabati is Jebel Sabaloka, located in the Sabaloka Inlier, which is part of the 6th Nile Cataract around 80 km downstream from the confluence of the Blue and White Niles. Many of the known Meroitic-period remains are located to the southwest of the mountain slopes. The remains consist of simple stone structures and camp sites attributed by the excavators to relatively mobile agro-pastoralists (Suková and Cílek 2012). There and elsewhere are clusters of Meroitic and post-Meroitic tumuli, some up to 9 m in diameter. Burials marked by cairns also occur on terraces in nearby wadis, which also contained settlements. No tumuli are present at Jebel Moya, where the graves differ in design. The inlier system of wadis, freshwater features, periodic swamps, constricted riverine landscape and hills made for a dynamic zone of interaction between peoples from the heart of the Meroitic state and agro-pastoralists exploiting both its ecological resources and security, and the outlier desert environment.

While various forms of burial were present in the Shendi Reach, their nature and structure is different to that of the burials at Jebel Moya and also that of the known tumuli in the southern Atbai. Rather than grounding explanations of variability in chronological or biological/genetic distinctions (Addison 1949), it seems more fruitful to explain the observable variation in practices in terms of complex, contextually situated social behaviours. The Jebel Moya pastoralists shared their land between the two Niles with agro-pastoralists and Meroitic trading partners. Their choice of Jebel Moya as a burial ground was a visible symbol, inscribing their ideological practices in a culturally varied landscape. By contrast, the southern Atbai—outside of the Gash Delta—was, to the best of our current knowledge, inhabited exclusively by nomads. Despite these differences, the southern Gezira and southern Atbai saw the growth of alternative but contemporary modes of economic exploitation and power bases within a wider set of interwoven social networks.

## Discussion

**Fig. 6:**
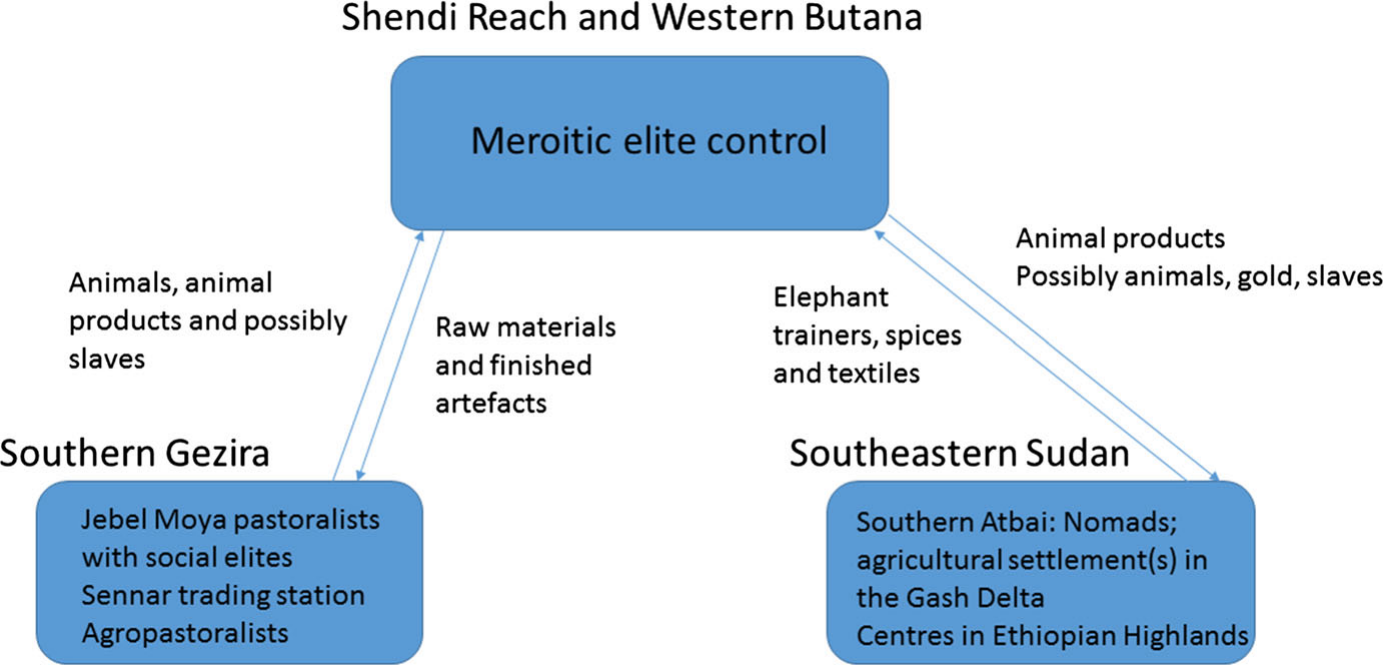
Synthesis figure of the proposed model

The establishment of state presence in areas where ecological conditions and trading opportunities permit is known to result in certain types of infrastructure, for example networks of bore wells, which have given rise to specialised highly mobile pastoralism, examples of which would be the Himba in Namibia (Bollig et al. 2013) and the mix of agricultural, agro-pastoral and pastoral communities in the Western Butana after the southward expansion of the Meroitic state into the region from the late first millennium BC onwards (Ahmed 1984; Bradley 1986, 1992). (The late first millennium BC is likely the time when the first *hafirs*—water dam-like structures—were constructed; the question of when the Gezira *hafirs* were first conceived is unexplored.) The model proposed in this paper recognises such events (Fig. 6); it also aligns with Linseele’s (2010) and Sadr’s (1991) models, which proposed that nomadic pastoralism first arose in Northeast Africa during the first millennium BC, for the southern Atbai (see Gatto 2014; Hafsaas 2006 for possible earlier, more northerly instances in the Eastern Desert). However, it differs in hypothesising that internal socio-political, rather than ecological or external political powers, were the cause of the establishment of (1) nomadic pastoralism in the southern Atbai, and (2) both agro-pastoralism and pastoralism in the southern Gezira. It should not be assumed that pastoralism arose in both areas simply because of the nature of interactions with the state, or that higher levels of social complexity were a consequence of relations with an established state. Instead, inter-cultural contact was nuanced, and led to the pastoralists of the southern Gezira and nomads of the southern Atbai structuring their lives in various ways through investment in their herds and their movements in the landscape.

The different trajectories of cultural evolution have been illustrated for the southern Gezira and the southern Atbai along the southern and southeastern frontier zones of the Meroitic state. For the former, the burials of pastoral peoples were relatively poor in terms of assemblages, had only a generalised orientation and lacked many discernible features of hierarchical leadership except for the occurrence of a spatial neighbourhood around 27 richer burials. The Jebel Moya massif is such a focal point in the physical landscape that the cemetery must have been a central symbolic focus too in the world of the pastoralists who were buried there, while petrographic analysis confirms for the first time that it was likely the Jebel Moya inhabitants who exploited and left behind the archaeological traces at Jebel Saqadi.

For both the southern Gezira and the southern Atbai, different forms of engagement by the Meroitic state had a direct impact upon power relations within the local areas. The southern Atbai was dominated by nomads, with a plausible area of agricultural production in the Gash Delta. It may have been a conduit for movement of craftspeople and perishable items. Here, Salzman’s (1972) multi-resource and multi-purpose model may apply. The pursuit of nomadism provided adaptability for the inhabitants in dryer ecological conditions and mobility was advantageous in their interactions with their neighbours. This flexibility in constructing alliances and networks over large distances shows political nuance in dealing with a variety of situationally different social structures and political expectations.

At the same time, little is known about the outbound flow of prestige goods from the Shendi Reach to the local populace in the southern Gezira. Arguably one of the best examples is provided by the carinated bronze bowls at Abu Geili, which are similar to those found in elite burials at Meroe (Addison 1950), reinforcing the elite exchange-network hypothesis of Edwards (1996). By contrast, the inbound flow of tribute and trade items to the Meroitic centre, from where they were redistributed to territories down the Nile, is better known: skins, ivory, gold, ostrich feathers and slaves. Although the extent, nature and localities of previously proposed gold extraction along the Blue Nile are as yet undetermined (Addison 1950; Dixon 1963), this does not have to signify direct exploitation: access to the resources could have been through intermediaries. If there was a Meroitic outpost at Sennar, then the areas to the west, including Jebel Moya, with its agro-pastoralist and pastoralist populations, could have acted as economic partners.

The reconstructed mix of economic systems in the southern Gezira and southern Atbai, and possible trade networks stretching to the Red Sea, necessitates a refocusing on frontier zones. Contacts between the Red Sea and the Nile Valley are known from earlier periods, particularly from the occurrence of cowrie beads (Linseele and Pöllath 2015), which are also present at Jebel Moya. It is the dynamic and possibly fluid nature of interaction between the state and local communities which is the enabler of social change, and these changes occur on both sides. Developing more sophisticated modelling of the subsequent social interactions and the social patterns in the material culture of the local communities, particularly pastoral societies, will assist us in getting to grips with interlocking micro-and regional-scale processes in the Sudan.
